# Start order and medal outcomes: An analysis of apparatus finals in men’s artistic gymnastics

**DOI:** 10.1371/journal.pone.0351760

**Published:** 2026-06-18

**Authors:** Chih-Yu Chen, Kuang-Hui Chen, Kang-Hao Lu

**Affiliations:** 1 Department of Physical Education, National Taitung University, Taitung, Taiwan; 2 Graduate Institute of Sports Science, National Taiwan Sport University, Taoyuan, Taiwan; 3 Taiwan Institute of Sports Science, Kaohsiung, Taiwan; Universiti Kebangsaan Malaysia Faculty of Medicine: Hospital Canselor Tuanku Muhriz UKM, MALAYSIA

## Abstract

**Purpose:**

This study examined the relationship between start order and competition outcomes in men’s artistic gymnastics apparatus finals.

**Methods:**

Competition outcomes from three Olympic Games and nine World Championships were analyzed. Ordinal-based analyses, including Spearman’s rank-order correlation and Kendall’s tau-b, were conducted to examine the relationship between start order and final rankings. Logistic regression was used to assess the association between start order and medal attainment. In addition, Mann–Whitney U tests were used to compare final rankings between start-order groups; independent-samples *t*-tests were used to assess differences in Difficulty (D) scores, Execution (E) scores, and total scores; and Chi-square tests were used to evaluate associations between start-order grouping, medal attainment, and continental affiliation.

**Results:**

Ordinal correlation and logistic regression analyses showed no significant associations between start order and final rankings or medal attainment across apparatuses. Start-order grouping had limited influence on final rankings across most apparatuses. A significant effect was observed only in the pommel horse, where second-half gymnasts achieved higher E-scores and won more medals. Medal distributions between European and non-European athletes were similar across apparatuses and start-order groupings.

**Conclusion:**

Overall, start-order grouping had minimal effects. However, pommel horse results highlight the importance of execution stability. The findings support the general fairness of the current judging system and Code of Points. Future research should examine warm-up procedures under revised FIG regulations and extend analyses to women’s artistic gymnastics.

## Introduction

The Olympic Games, which are held every 4 years, are a global multisport event with more than 100 years of history. Artistic gymnastics has evolved into one of the most popular Olympic events. The Olympic Games represent one of the most prestigious artistic gymnastics competitions. Athletes must be qualified to participate, and only the world’s top gymnasts can participate in Olympic artistic gymnastics events. The Artistic Gymnastics World Championships (hereafter referred to as the World Championships) are regarded as the highest-level event for artistic gymnastics apparatus competitions and also serve as an important qualifying event for the Olympic Games. Men’s artistic gymnastics comprises six apparatuses: floor exercise, pommel horse, still rings, vault, parallel bars, and horizontal bar. Male gymnasts must perform different skills of varying difficulty on each apparatus [1]. The competition structure of both the Olympic Games and the World Championships includes the qualification round, team final, all-around final, and apparatus finals. According to the Code of Points for Men’s Artistic Gymnastics, eligibility for the team final, all-around final, and apparatus finals is determined by the results of the qualification round. Only the teams or individual athletes who advance to the finals have the opportunity to compete for medals [2].

The apparatus finals involve the top eight athletes for each apparatus from the qualification round. Each finalist performs one complete routine, and the final ranking is determined on the basis of the total score obtained. Only the top three athletes for each apparatus receive medals (gold, silver, and bronze). The final score for each athlete is the sum of the Difficulty score (i.e., the D-score) and the Execution score (i.e., the E-score). The D-score comprises the difficulty value, element values, composition requirements, and connection value. According to the 2013–2024 Code of Points, the D-panel judges select the 10 most difficult elements (including the dismount) performed by the gymnast and sum their corresponding element values to calculate the total D-score. By contrast, in the vault event, the D-score is determined by the difficulty value of a single vault, which differs from the calculation methods used for other apparatuses. In addition to calculating D-scores, the D-panel judges also impose neutral deductions for faults such as stepping out of bounds (on floor exercise and vault) or exceeding the allotted time during the routine. For all six apparatuses, the E-score begins at 10.0, from which deductions are made for execution faults. The E-panel judges evaluate the gymnast’s routine by deducting points for technical errors, deviations from body line standards, or performance mistakes. Kerwin and Irwin [3] noted that gymnasts must achieve an optimal balance between D-score and E-score performance to achieve higher total scores and rankings.

After qualifying for the apparatus finals, gymnasts perform their complete routines in a certain order, which is determined on the basis of start order. The start order for every apparatus final is randomly assigned by draw; thus, it varies from competition to competition. The following studies have demonstrated that start order significantly affects judging outcomes. Wilson [4] reported that athletes’ order of appearance in competitions affected their scoring results, noting that athletes who performed later tended to receive higher scores than those who performed earlier. Damisch et al. [5] observed a positive correlation between start order and final score, suggesting the presence of an overall sequential-order bias. Rotthoff [6] argued that athletes who competed later generally have an advantage in E-score received. Collectively, these studies have indicated that gymnasts performing later in the apparatus finals are more likely to achieve higher rankings. From a practical standpoint, coaches and athletes tend to believe that performing later provides several advantages. Later competitors have more time to prepare and can observe the routines of earlier gymnasts, allowing them to adjust their competition strategies [7]. National bias has been observed in numerous sports that rely on subjective judging, including figure skating, artistic gymnastics, diving, and boxing [8]. Taken together, the aforesaid findings suggest that once athletes enter the apparatus finals, their final results may be affected by not only their performance strategies but also start-order effects and judging tendencies. Therefore, these phenomena merit further exploration to examine how start order affects athletes’ final rankings and whether differences in national or continental affiliation lead to variations in judging outcomes during apparatus finals.

On the basis of the foregoing discussion, start order may affect athletes’ E-score performance in apparatus finals. Whether start order also affects athletes’ likelihood of winning medals has not yet been thoroughly examined. Therefore, this study compiled and analyzed data by grouping finalists for each apparatus on the basis of start order. Additionally, after summarizing the national distribution of finalists across apparatuses, the data were further categorized by continent into two groups, namely the European and non-European groups, to examine whether the relationship between medal outcomes and start order varied with the regional affiliation of athletes and to determine whether any ranking bias related to continental affiliation existed. Accordingly, this study conducted an in-depth analysis of the relationship between start order, final ranking, and medal attainment among male artistic gymnasts who competed in the apparatus finals of three Olympic Games and nine World Championships (a total of 12 competitions) held between 2013 and 2024. The objectives of this study were (1) to examine the relationship between different start-order groupings and final rankings in each apparatus event; (2) to compare D-scores, E-scores, and total scores among gymnasts in different start-order groups; (3) to analyze the relationship between start-order grouping and medal outcomes across apparatus finals; and (4) to explore the relationship between start-order grouping and medal outcomes among European and non-European athletes.

## Materials and methods

### Participants

The participants of this study were male artistic gymnasts who competed in the six apparatus finals events, namely floor exercise, pommel horse, still rings, vault, parallel bars, and horizontal bar, across three Olympic Games and nine World Championships held between 2013 and 2024 (a total of 12 competitions). Qualification for each apparatus final was granted to the top eight athletes in the respective qualification rounds, with a maximum of two gymnasts per country permitted to advance. In instances where the gymnast ranked ninth in the qualification round had the same D-score, E-score, and final score as the eighth-ranked gymnast, both were allowed to progress to the final. Consequently, some apparatus finals included a ninth competitor. In total, data were collected from 580 finalist appearances. All participants were senior male elite gymnasts who qualified for apparatus finals according to FIG regulations. Because the study relied on publicly available competition records, individual demographic variables such as exact age, educational background, and socioeconomic status were not accessible. Additional demographic and competition characteristics of the study sample are presented in S1 Table.

### Data collection

Data were obtained from the apparatus finals of three Olympic Games, namely Rio 2016, Tokyo 2020, and Paris 2024, and nine World Championships in men’s artistic gymnastics. The years and locations of the World Championships are as follows: Antwerp (2013); Nanning (2014); Glasgow (2015); Montreal (2017); Doha (2018); Stuttgart (2019); Kitakyushu (2021); Liverpool (2022); and Antwerp (2023). Performance records for all apparatus finals were obtained from publicly accessible official competition records published on the FIG/World Gymnastics official results platform. The FIG Event IDs and direct URLs for all competitions included in this study are provided in S2 Table. On each official event page, the official result files can be accessed by clicking “Show Files” under “Event Files” and selecting the relevant official result files, such as results books, start lists, qualification results, final results, and related competition records.

### Data processing

The official competition results from the 2016, 2020, and 2024 Olympic Games and the 2013–2023 World Championships included the start order, nationality, and final ranking of male gymnasts in each apparatus final. These data were directly used in the compilation and statistical analyses for this study. The data compilation procedure is as follows:

(1) The data entry sequence began with the three Olympic Games (2016 Rio, 2020 Tokyo, and 2024 Paris), followed by the World Championships in chronological order: 2013 (Antwerp), 2014 (Nanning), 2015 (Glasgow), 2017 (Montreal), 2018 (Doha), 2019 (Stuttgart), 2021 (Kitakyushu), 2022 (Liverpool), and 2023 (Antwerp).
(2) Data for the apparatus events were recorded in the following order: floor exercise, pommel horse, still rings, vault, parallel bars, and horizontal bar.
(3) The following information was recorded for each apparatus final: start order, country represented, final ranking, D-score, E-score, and total score. Data for each final were compiled beginning with the first-place gymnast, followed sequentially through to the last-ranked finalist. All data were entered into Microsoft Excel 2016 (Microsoft Corp., Redmond, WA, USA).
(4) After entry, each gymnast’s data, including start order, country, final ranking, D-score, E-score, and total score, were cross-checked against the information published on the official website to ensure accuracy before proceeding to enter the data of the next gymnast. In the vault final, each gymnast performed two different vaults, each receiving its own D-score, E-score, and final score. The D-scores and E-scores from the two vaults were averaged separately, and the final score was defined as the average of the two vault scores.
(5) Upon verifying the accuracy of all gymnast data for a given apparatus and competition, the same cross-checking procedure was repeated for the next apparatus. This process was applied consistently across all events and competitions.
(6) Once all data were recorded, gymnasts were grouped in accordance with start order: athletes who performed in orders 1–4 were categorized as the “first-half group,” and those in orders 5–8 (or 5–9 when applicable) comprised the “second-half group.” Examining the distribution of finalists’ national affiliations (Table 1) revealed that athletes from European countries constituted the largest proportion (*n* = 281), followed by Asia (*n* = 195), the Americas (*n* = 100), Oceania (*n* = 4), and Africa (*n* = 0). Accordingly, the dataset was divided into two geographic groups, namely the European and non-European groups, with all European nations classified as the former and all Asian, American, Oceanian, and African countries classified as the latter.

**Table 1:** Distribution of finalists’ national affiliations by apparatus.

	Floor exercise	Pommel horse	Still rings	Vault	Parallel bars	Horizontal bar	Total
**Europe**	36	56	64	51	41	33	281
**Asia**	41	28	22	31	39	34	195
**America**	21	12	10	14	17	26	100
**Oceania**	0	1	0	0	0	3	4
**Africa**	0	0	0	0	0	0	0
**Total**	98	97	96	96	97	96	580

Note: Data represent number of finalists.

### Data analyses

After data collection and processing were completed, statistical analyses were conducted using SPSS Statistics 24.0 for Windows (IBM Corp., Armonk, NY, USA). To comprehensively examine the effect of start order, both ordinal-based analyses and grouping-based analyses were performed.

(1) Spearman’s rank-order correlation and Kendall’s tau-b correlation analyses were conducted to examine the relationships between start order (positions 1–8 or 1–9 in the case of ties) and final rankings across apparatus finals.
(2) Binary logistic regression analyses were performed to examine the association between start order and medal outcomes (medaled vs. nonmedaled) for each apparatus. Odds ratios (Exp(B)) and their corresponding 95% confidence intervals were reported.
(3) The Mann–Whitney U test (nonparametric) was used to compare the final ranking distribution between the two start-order groups for each apparatus each year, namely floor exercise, pommel horse, still rings, vault, parallel bars, and horizontal bar. The rank-biserial correlation (r_rb_) was used to indicate effect size, which ranged from −1–1; larger values indicate greater effect sizes.
(4) Chi-square tests were used to examine the association between start-order grouping and medal outcomes (medaled vs. nonmedaled) for athletes in each apparatus final. Cramér’s V was used to indicate effect size.
(5) Independent-samples *t* tests were conducted to compare D-scores, E-scores, and total scores in the two start-order groups for each apparatus. Cohen’s d was used to indicate effect size.
(6) Chi-square tests were also used to compare the association between start-order grouping and medal outcomes among European and non-European athletes across all six apparatus events. Cramér’s V was used to indicate effect size. In addition to testing for statistical significance, this study also reported effect sizes to assess the magnitude of practical effects. The level of statistical significance was set at α = 0.05.

### Ethical considerations

This study was based exclusively on publicly available official competition results published on the FIG/World Gymnastics official results platform. The data included only competition-related variables, such as start order, final ranking, D-score, E-score, final score, apparatus, competition year, nationality, and medal outcome. No private, confidential, nonpublic, or personally sensitive information was collected or analyzed. Therefore, institutional ethical approval and informed consent were not required.

## Results

### Associations between start order and final rankings and medal outcomes across apparatus finals

As shown in Table 2, no significant associations were observed between start order and either final rankings or medal outcomes across all apparatus. Kendall’s tau-b and Spearman’s rho indicated no ordinal relationships (all *p* > 0.05). However, in the parallel bars event, although the association did not reach statistical significance, the results approached the threshold of significance (Spearman’s ρ = 0.194, *p* = 0.057), suggesting a potential trend. Logistic regression analyses further indicated that start order was not significantly associated with medal attainment (all *p* *>* 0.05).

**Table 2:** Associations between start order and final rankings and medal outcomes.

Apparatus	Kendall’s τb	*p*	Spearman’s ρ	*p*	Exp(B)	95% CI	*p*
**Floor exercise**	0.015	0.847	0.025	0.810	1.011	0.847–1.207	0.904
**Pommel horse**	−0.102	0.183	−0.145	0.157	1.155	0.963–1.385	0.120
**Still rings**	0.102	0.184	0.133	0.197	0.910	0.759–1.092	0.313
**Vault**	−0.089	0.246	−0.117	0.256	1.070	0.893–1.283	0.463
**Parallel bars**	0.147	0.054	0.194	0.057	0.970	0.813–1.159	0.740
**Horizontal bar**	0.113	0.141	0.155	0.132	0.923	0.771–1.106	0.386

Note: τb = Kendall’s tau-b; ρ = Spearman’s rho; Exp(B) = odds ratio; CI = confidence interval. Final rankings were analyzed using ordinal correlation, whereas medal outcomes were analyzed using logistic regression (0 = no medal, 1 = medal). No significant differences were observed across all apparatus (*p* > 0.05).

### Effects of start-order grouping on final rankings across apparatuses

**Fig 1:**
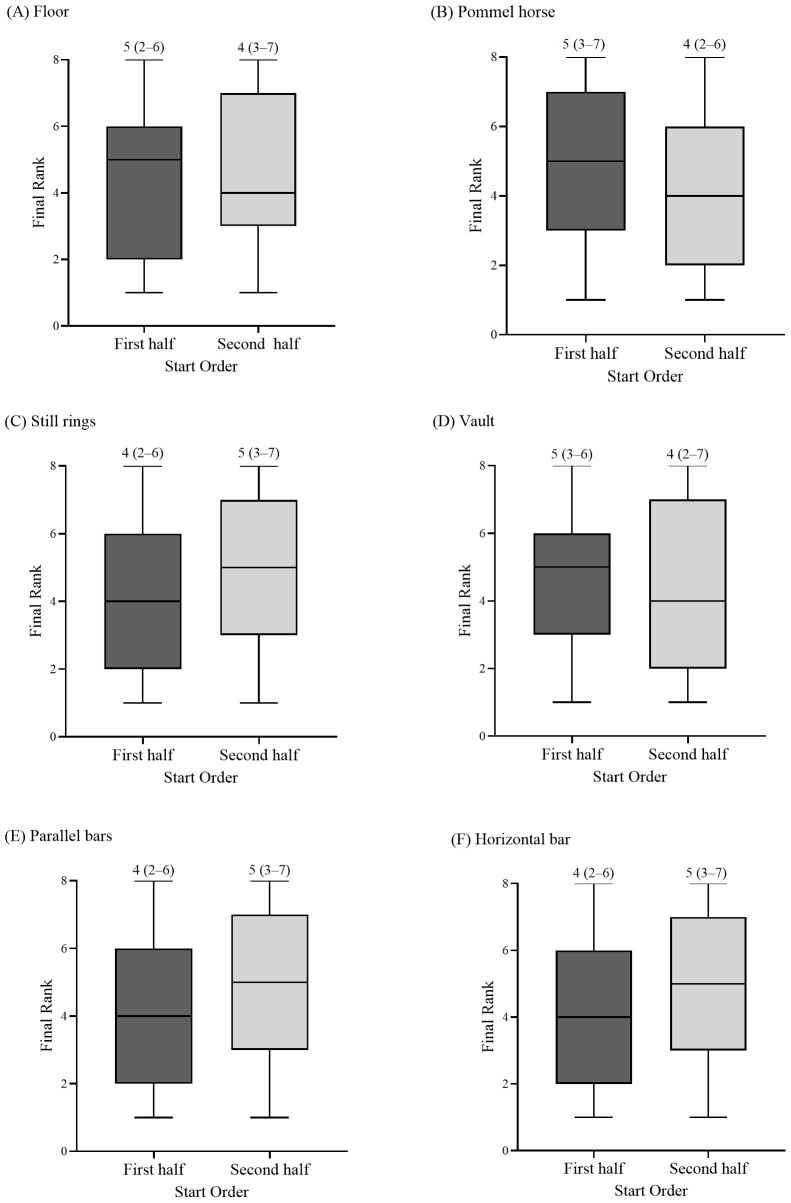
Comparison of final ranking distributions between start-order groups across six apparatus finals. Note. Box-and-whisker plots illustrate the distribution of final rankings for the first-half and second-half groups across six apparatus finals: (A) floor exercise, (B) pommel horse, (C) still rings, (D) vault, (E) parallel bars, and (F) horizontal bar. Boxes represent the interquartile range (Q1–Q3), the horizontal line within each box indicates the median, and whiskers denote the minimum and maximum values. No significant differences were observed between the two groups across all apparatus (*p* > 0.05).

No significant differences in rankings were observed between the two start-order groups in any of the six apparatus finals (Fig 1); *p* values were 0.400 for floor exercise, 0.081 for pommel horse, 0.184 for still rings, 0.929 for vault, 0.075 for parallel bars, and 0.122 for horizontal bar. Accordingly, the final rankings of gymnasts in apparatus finals of the 12 competitions were generally not affected by start-order grouping. As shown in Fig 1, no significant differences were observed between start-order groups and ranking performance in floor exercise (*p* = 0.398, r_rb_ = 0.09), pommel horse (*p* = 0.081, r_rb_ = 0.21), still rings (*p* = 0.184, r_rb_ = 0.16), vault (*p* = 0.929, r_rb_ = 0.01), parallel bars (*p* = 0.075, r_rb_ = 0.20), and horizontal bar (*p* = 0.122, r_rb_ = 0.18). The median rankings and interquartile ranges for each apparatus are presented in Fig 1. Overall, effect sizes ranged from 0.01 to 0.21 across apparatuses (i.e., from negligible to small effects). These findings indicate that final rankings across the 12 apparatus finals included in this study were minimally influenced by start-order grouping.

### Medal outcomes by start-order grouping across apparatuses

As shown in Table 3, Chi-square tests were conducted to examine the association between start-order grouping and medal attainment across the six apparatus events (i.e., floor exercise, pommel horse, still rings, vault, parallel bars, and horizontal bar). A significant association was observed only in the pommel horse event (χ²(1) = 4.096, *p* = 0.043, Cramér’s V = 0.205). Specifically, in the pommel horse final, athletes in the first-half group recorded 13 medal-winning performances and 35 nonmedaled performances, whereas those in the second-half group recorded 23 medal-winning performances and 26 nonmedaled performances, showing a higher proportion of medal attainment among athletes competing in the second-half. However, the effect size (Cramér’s V = 0.205) did not reach the threshold for a medium effect, suggesting that although the association was significant, its practical magnitude was limited. No significant associations were found for floor exercise (χ²(1) = 0.024, *p* = 0.878, Cramér’s V = 0.016), still rings (χ²(1) = 2.844, *p* = 0.092, Cramér’s V = 0.208), vault (χ²(1) = 0.178, *p* = 0.673, Cramér’s V = 0.043), parallel bars (χ²(1) = 0.248, *p* = 0.618, Cramér’s V = 0.051), or horizontal bar (χ²(1) =1.099, *p* = 0.294, Cramér’s V = 0.107). The corresponding effect sizes were predominantly negligible to small, indicating a generally weak association between start-order grouping and medal outcomes.

**Table 3:** Association between start-order grouping and medal outcomes in men’s apparatus finals.

Apparatus	Group	Medaled (%)	Nonmedaled (%)	χ²	*p*	Cramér’s V
**Floor exercise**	First-half group	18 (37.5%)	30 (62.5%)	0.024	0.878	0.016
	Second-half group	18 (36.0%)	32 (64.0%)			
**Pommel horse**	First-half group	13 (27.1%)	35 (72.9%)	4.096	0.043^*^	0.205
	Second-half group	23 (46.9%)	26 (53.1%)			
**Still rings**	First-half group	22 (45.8%)	26 (54.2%)	2.844	0.092	0.208
	Second-half group	14 (29.2%)	34 (70.8%)			
**Vault**	First-half group	19 (39.6%)	29 (60.4%)	0.178	0.673	0.043
	Second-half group	17 (35.4%)	31 (64.6%)			
**Parallel bars**	First-half group	19 (39.6%)	29 (60.4%)	0.248	0.618	0.051
	Second-half group	17 (34.7%)	32 (65.3%)			
**Horizontal bar**	First-half group	21 (43.8%)	27 (56.3%)	1.099	0.294	0.107
	Second-half group	17 (34.7%)	32 (65.3%)			

*Note*: χ² = Chi-square; *df* = degree of freedom; Cramér’s V = effect size. Percentages represent within-group proportions. All comparisons were based on 2 × 2 contingency tables (*df* = 1). ^*^ *p* < 0.05.

As shown in Table 4, among the men’s apparatus finals, a significant difference in E-score was observed only in the pommel horse event. Specifically, the mean E-score was significantly higher in the second-half group (8.41 ± 0.59) than in the first-half group (8.07 ± 0.79; *p* = 0.017). However, the effect size (Cohen’s d ≈ 0.49) did not reach the threshold for a large effect, indicating that although the difference was significant, its practical magnitude was limited. By contrast, no significant differences were found in D-scores (first-half: 6.50 ± 0.42 vs. second-half: 6.49 ± 0.36, *p* = 0.918) or total scores (first-half: 14.57 ± 1.07 vs. second-half: 14.90 ± 0.81, *p* = 0.089). These results indicate that athletes in the two start-order groups exhibited similar performance in terms of D-scores and total scores in the pommel horse final. Although the difference in total scores did not reach significance, the *p* value was close to the significance threshold (*p* = 0.089).

**Table 4:** D-scores, E-scores, and total scores stratified by start-order group for each apparatus event.

Apparatus	Score	First-half group (M ± SD)	Second-half group (M ± SD)	*t*	*p*	d
**Floor exercise**	D-score	6.41 ± 0.36	6.42 ± 0.43	−0.043	0.966	−0.009
	E-score	8.29 ± 0.49	8.28 ± 0.49	0.055	0.956	0.011
	Total score	14.70 ± 0.65	14.70 ± 0.70	0.015	0.988	0.003
**Pommel horse**	D-score	6.50 ± 0.42	6.49 ± 0.36	0.103	0.918	0.021
	E-score	8.07 ± 0.79	8.41 ± 0.59	−2.419	0.017^*^	−0.491
	Total score	14.57 ± 1.07	14.90 ± 0.81	−1.719	0.089	−0.349
**Still rings**	D-score	6.45 ± 0.32	6.40 ± 0.31	0.714	0.477	0.146
	E-score	8.59 ± 0.40	8.65 ± 0.24	−0.899	0.371	−0.183
	Total score	15.04 ± 0.55	15.05 ± 0.42	−0.139	0.890	−0.028
**Vault**	D-score	5.75 ± 0.31	5.71 ± 0.29	0.690	0.492	0.141
	E-score	9.02 ± 0.37	9.02 ± 0.35	0.006	0.995	0.001
	Total score	14.77 ± 0.40	14.71 ± 0.51	0.564	0.574	0.115
**Parallel bars**	D-score	6.61 ± 0.32	6.48 ± 0.37	1.854	0.067	0.377
	E-score	8.53 ± 0.60	8.50 ± 0.59	0.313	0.755	0.063
	Total score	15.14 ± 0.73	14.97 ± 0.84	1.043	0.299	0.212
**Horizontal bar**	D-score	6.45 ± 0.53	6.32 ± 0.58	1.137	0.259	0.232
	E-score	7.96 ± 0.66	7.85 ± 0.78	0.694	0.490	0.142
	Total score	14.41 ± 0.95	14.18 ± 1.17	1.066	0.289	0.218

Note: M = mean; SD = standard deviation; *t* = independent-samples *t*-test; *d* = Cohen’s d effect size. For floor exercise, first-half group *n* = 48, and second-half group *n* = 50; for parallel bars, second-half group *n* = 49; for all other apparatuses, first-half group *n* = 48, and second-half group = 49–50, depending on the apparatus. ^*^p < 0.05.

In the remaining five apparatus finals, namely floor exercise, still rings, vault, parallel bars, and horizontal bar, no significant differences were observed between the first-half and second-half groups in D-score, E-score, or total score. For floor exercise, the D-scores were 6.41 ± 0.36 for the first-half group and 6.42 ± 0.43 for the second-half group (*p* = 0.966); the E-scores were 8.29 ± 0.49 and 8.28 ± 0.49, respectively (*p* = 0.956); and the total scores were 14.70 ± 0.65 and 14.70 ± 0.70, respectively (*p* = 0.988). For still rings, the D-scores were 6.45 ± 0.32 for the first-half group and 6.40 ± 0.31 for the second-half group (*p* = 0.477); the E-scores were 8.59 ± 0.40 and 8.65 ± 0.24, respectively (*p* = 0.371); and the total scores were 15.04 ± 0.55 and 15.05 ± 0.42, respectively (*p* = 0.890). For vault, the D-scores were 5.75 ± 0.31 for the first-half group and 5.71 ± 0.29 for the second-half group (*p* = 0.492); the E-scores were 9.02 ± 0.37 and 9.02 ± 0.35, respectively (*p* = 0.995); and the total scores were 14.77 ± 0.40 and 14.71 ± 0.51, respectively (*p* = 0.574). In the parallel bars event, the D-scores were 6.61 ± 0.32 for the first-half group and 6.48 ± 0.37 for the second-half group (*p* = 0.067); the E-scores were 8.53 ± 0.60 and 8.50 ± 0.59, respectively (*p* = 0.755); and the total scores were 15.14 ± 0.73 and 14.97 ± 0.84, respectively (*p* = 0.299). For horizontal bar, the D-scores were 6.45 ± 0.53 for the first-half group and 6.32 ± 0.58 for the second-half group (*p* = 0.259); the E-scores were 7.96 ± 0.66 and 7.85 ± 0.78, respectively (*p* = 0.490); and the total scores were 14.41 ± 0.95 and 14.18 ± 1.17, respectively *(p* = 0.289). These results revealed that start-order grouping had limited effects on the performance outcomes in these apparatus events.

### Association between start-order grouping and medal outcomes among European and non-European athletes

As shown in Table 5, Chi-square tests were conducted to examine the association between start-order grouping and medal attainment among European and non-European male gymnasts in apparatus finals. The results indicated no significant differences in floor exercise (χ²(1) = 2.857, *p* = 0.091, Cramér’s V = 0.282), pommel horse (χ²(1) = 1.541, *p* = 0.214, Cramér’s V = 0.207), still rings (χ²(1) = 0.286, *p* = 0.593, Cramér’s V = 0.089), vault (χ²(1) = 0.111, *p* = 0.738, Cramér’s V = 0.056), parallel bars (χ²(1) = 0.003, *p* = 0.955, Cramér’s V = 0.009), and horizontal bar (χ²(1) = 0.711, *p* = 0.399, Cramér’s V = 0.139), with all *p* values exceeding 0.05. In terms of effect size, Cramér’s V ranged from 0.009 to 0.282 across apparatuses, signifying small-to-moderate effects. These findings suggest that start-order grouping did not clearly influence medaling outcomes for either European or non-European athletes.

**Table 5:** Association between start-order grouping and medal outcomes among European and Non-European athletes.

Apparatus	Group	European (%)	Non-European (%)	χ²	*p*	Cramér’s V
**Floor exercise**	First-half group	10 (55.6%)	8 (44.4%)	2.857	0.091	0.282
	Second-half group	5 (27.8%)	13 (72.2%)			
**Pommel horse**	First-half group	9 (69.2%)	4 (30.8%)	1.541	0.214	0.207
	Second-half group	11 (47.8%)	12 (52.2%)			
**Still rings**	First-half group	13 (59.1%)	9 (40.9%)	0.286	0.593	0.089
	Second-half group	7 (50.0%)	7 (50.0%)			
**Vault**	First-half group	9 (47.4%)	10 (52.6%)	0.111	0.738	0.056
	Second-half group	9 (52.9%)	8 (47.1%)			
**Parallel bars**	First-half group	8 (42.1%)	11 (57.9%)	0.003	0.955	0.009
	Second-half group	7 (41.2%)	10 (58.8%)			
**Horizontal bar**	First-half group	8 (38.1%)	13 (61.9%)	0.711	0.399	0.139
	Second-half group	4 (25.0%)	13 (75.0%)			

Note: χ² = Chi-square; df = degree of freedom; Cramér’s V = effect size. Percentages represent within-group proportions. All comparisons were based on 2 × 2 contingency tables (*df* = 1). ^*^ *p* < 0.05.

## Discussion

### Effects of start-order on final rankings

As shown in Table 2, correlation analyses indicated that start order was not significantly associated with final rankings across any apparatus, suggesting that start order did not exert a clear influence on gymnasts’ final placements in apparatus finals. Further analyses examining the relationship between different start-order groups and final rankings demonstrated that the nonparametric tests revealed no significant differences in final rankings between start-order groups across all six apparatus finals (Fig 1). In addition, the effect sizes (r_rb_) across apparatuses ranged from −0.010 to 0.208 (i.e., from negligible to small). This indicated that start-order differences exerted limited influence on final rankings in men’s artistic gymnastics apparatus finals. Rotthoff [6] reported that in women’s artistic gymnastics apparatus finals, athletes performing later in the start order achieved significantly higher E-scores; specifically, each subsequent start position was associated with an average increase of approximately 0.01 to 0.03 points in E-score. This finding suggests that scores are positively correlated with start order. Pizzera [9] noted that in aesthetic sports, scoring decisions may be influenced by the cognitive biases of judges and the performances of preceding athletes, such that evaluations are not based entirely on objective criteria. Suominen [10] further stated that in high-level artistic gymnastics competitions, even minimal score differences may alter final rankings. Overall, the research suggests that start order indirectly affects final rankings by influencing scoring outcomes. However, the present study found no significant association between start order and final rankings in men’s artistic gymnastics. Joustra et al. [11] obtained similar findings, reporting no overall order effects in men’s events. Rotthoff [12] remarked that substantial differences exist between men’s and women’s artistic gymnastics in terms of scoring systems, particularly with respect to artistic evaluation. In addition to technical elements, women’s artistic gymnastics includes artistic components such as rhythm, musical interpretation, and choreography, rendering the scoring process more subjective. Men’s artistic gymnastics places greater emphasis on technical difficulty and execution quality [2,13]. Given differences in scoring criteria and the random assignment of start order by draw in apparatus finals, the influence of start order on final rankings in men’s artistic gymnastics is likely to be relatively limited.

### Relationship between start-order and medal outcomes

As shown in Table 2, logistic regression analyses indicated that start order was not significantly associated with medal outcomes across any apparatus, suggesting that start order did not exert a significant influence on medal attainment. Further analyses comparing medal outcomes between different start-order groups revealed that gymnasts competing in the second-half group won significantly more medals than those in the first-half group did in the pommel horse final (Table 3). Furthermore, the average E-score of gymnasts in the second-half group was significantly higher than that of their first-half counterparts (Table 4). These results may reflect a relative advantage for athletes in the second-half group in terms of execution performance. However, because the effect size did not reach the threshold for a large effect, the magnitude of this influence appears to be limited. Prassas et al. [14] informed that pommel horse requires athletes to maintain balance under dynamic conditions while executing skills, making the apparatus one of the most technically demanding in men’s artistic gymnastics. During pommel horse routines, gymnasts are required to perform continuous circular movements (with legs together or straddled) on the apparatus. These movements involve rotation of the body’s center of mass around the support point and rotation about both the vertical and longitudinal axes. Consequently, even minor deviations in movement can lead to a loss of balance or a fall [15]. In accordance with the Code of Points, point deductions are required for hitting the apparatus, pausing during a routine, or falling [2]. A single major fault, such as a prolonged pause, fall, or strong collision with the apparatus, can eliminate a gymnast’s chance of medaling. Kalinski et al. [16] noted that when athletes have similar D-scores, the E-score becomes the deciding factor affecting the final score. Therefore, athletes in the second-half group may demonstrate greater routine stability and higher success rates, resulting in superior E-scores that may be associated with a higher frequency of medal attainment. However, because the effect size did not reach a moderate level, the practical significance of this association remains limited. Taken together, these findings suggest that although a clear association between start-order grouping and medal outcomes exists in the pommel horse event, start-order grouping does not exert a substantial influence on rankings or score composition in the floor exercise, still rings, vault, parallel bars, or horizontal bar events. Cervin [17] argued that the 2006 revision of the International Code of Points, which separated the D-score and E-score, increased the competitive advantage of athletes performing higher-difficulty routines. Increasing the D-score is a key strategy for athletes to improve their final scores in each apparatus, and the overall score is closely associated with medal attainment [18]. Chen et al. [19] investigated whether increasing the D-score in apparatus finals improves an athlete’s chance of earning a medal, finding that this approach was particularly effective in the pommel horse and horizontal bar events. Overall, the findings from relevant studies may inform the development of routine composition strategies and the strategic use of start order across different apparatus events to improve medaling outcomes.

Furthermore, different statistical approaches may vary in their sensitivity to detecting start-order effects. Analyses that preserve the ordinal nature of start order are more suitable for capturing overall trends, whereas grouping approaches may better reflect structural differences under specific competition formats. Therefore, future studies are encouraged to consider both original start-order positions and grouping strategies to provide a more comprehensive understanding of start-order effects on performance outcomes.

### Relationship between start-order grouping and medal outcomes among European and Non-European athletes

As shown in Table 5, no significant differences were observed in medal outcomes between European and non-European athletes across start-order groups in the apparatus finals for floor exercise, pommel horse, still rings, vault, parallel bars, or horizontal bar. These findings indicate that medal attainment did not differ meaningfully between European and non-European finalists across different start-order groupings. Another study highlighted that judges may assign higher scores to athletes from their own country [7]. In addition, research has shown that the extent of national bias may vary among judges from different countries, indicating that scoring may be influenced by national or regional factors. However, across the 12 competitions analyzed in this study, no significant differences were observed in medal outcomes between European and non-European athletes across start-order groups. Sandberg [20] reported that judging decisions may be influenced by multiple sources of bias, including national bias. Other studies have maintained that the magnitude of such biases is generally limited and can be mitigated through the design of scoring systems and the implementation of judge monitoring mechanisms [21]. The scoring rules for men’s artistic gymnastics are revised following each Olympic cycle [2]. Notably, several judging controversies during the 2004 Athens Olympic Games prompted the FIG to implement substantial reforms to the scoring system, including the abolition of the traditional “perfect 10” system in 2006, with the aim of enhancing fairness and transparency [22]. Furthermore, adjustments to the composition of judging panels—such as excluding judges of the same nationality as the competing athlete or ensuring balanced national representation—may help reduce direct scoring bias [8]. With advances in technology, the FIG has also introduced the Computerised Gymnastics Judging Support System, which incorporates artificial intelligence and motion analysis techniques to assist judges in evaluating performance, thereby improving the objectivity and consistency of scoring [23]. Accordingly, revisions to scoring regulations, adjustments to judging panel composition, and the integration of technological support systems may collectively contribute to reducing bias among judges and enhancing overall scoring consistency.

### Limitations

This study has several limitations. First, according to FIG regulations, only eight athletes are permitted to advance to each apparatus final. Therefore, the sample size available for analyses within each individual competition was inherently limited. Although the present study included all available data from men’s artistic gymnastics apparatus finals at the Gymnastics World Championships and Olympic Games between 2013 and 2024, the overall sample size remained restricted by the design of finalist participation. Second, this study primarily analyzed final rankings, medal outcomes, and score performance. The study did not incorporate individual judges’ scoring data. As a result, directly examining judging bias at the individual judge level was not feasible. Third, the scoring rules for men’s artistic gymnastics are revised after each 4-year Olympic cycle. Because this study spans multiple cycles, the various versions of the Code of Points may involve different difficulty values and deduction criteria. This study did not differentiate between Olympic cycles. Future research could conduct more fine-grained comparative analyses across different Olympic cycles to better understand how changes in scoring regulations influence competition outcomes.

## Conclusions

This study yielded three primary findings. First, start-order grouping exerted little effects on athletes’ final rankings across apparatus finals. Second, start-order grouping affected medaling outcomes in the pommel horse event. In particular, athletes competing in the second-half group had a higher likelihood of winning a medal, and achieved higher E-scores, informing greater execution stability among second-half performers in the pommel horse final. Third, medal outcomes by athletes from different continental regions did not significantly differ by start-order grouping, indicating no evidence of continental bias in judging among high-level international judges. These findings support the conclusion that the current judging system is functioning fairly across geographic regions. Future research may further explore this topic by examining apparatus finals in women’s artistic gymnastics.

Prior to the Tokyo 2020 Olympic Games, athletes competing in apparatus finals were not provided with the opportunity to warm up on the competition apparatus. Once athlete introductions concluded, the first gymnast was required to begin the routine immediately. However, under the 2022–2024 Code of Points for men’s artistic gymnastics, the competition warm-up protocol was revised. Athletes ranked first through fourth in the start order are now permitted to warm up on the competition apparatus following athlete introductions and prior to the start of the first-half group. After the first-half routines conclude, athletes ranked fifth through eighth (or ninth if applicable) are allowed to warm up before the second-half group begins competition [2]. In the present study, only three competitions adopted this updated warm-up regulation. Therefore, whether this change in policy influences the effect of start order on final rankings remains inconclusive. Future studies may consider expanding the dataset and lengthening the observation period to further assess the effects of competition warm-up policies on performance outcomes and start-order effects in apparatus finals.

## Supporting information

S1 Data Raw dataset used in the present study. This file contains the dataset used for the statistical analyses, including apparatus, competition year, start order, final ranking, D-score, E-score, final score, nationality, and medal outcome.
(XLSX)

S1 Table Demographic and competition characteristics of the study samples. This table summarizes the demographic and competition characteristics of the study samples. The competitions included three Olympic Games—2016 (Rio), 2020 (Tokyo), and 2024 (Paris)—and nine World Championships: 2013 (Antwerp), 2014 (Nanning), 2015 (Glasgow), 2017 (Montreal), 2018 (Doha), 2019 (Stuttgart), 2021 (Kitakyushu), 2022 (Liverpool), and 2023 (Antwerp).
(DOCX)

S2 Table FIG/World Gymnastics event identifiers and direct URLs for competitions included in this study. This table provides the FIG Event IDs and direct URLs for the three Olympic Games and nine World Championships included in the present analysis. Each URL links to an official FIG/World Gymnastics event page. The official result files can be accessed from each event page by clicking “Show Files” under “Event Files.”.
(DOCX)
