# Supplementary material for: Start order and medal outcomes: An analysis of apparatus finals in men’s artistic gymnastics
Source: PLoS One. 2026 Jun 18;21(6):e0351760. doi: 10.1371/journal.pone.0351760 (PMC13278440; doi:10.1371/journal.pone.0351760)
Supplement: S1 Table — This table summarizes the demographic and competition characteristics of the study samples. The competitions included three Olympic Games—2016 (Rio), 2020 (Tokyo), and 2024 (Paris)—and nine World Championships: 2013 (Antwerp), 2014 (Nanning), 2015 (Glasgow), 2017 (Montreal), 2018 (Doha), 2019 (Stuttgart), 2021 (Kitakyushu), 2022 (Liverpool), and 2023 (Antwerp). (DOCX) [file pone.0351760.s002.docx]

| **S1 Table. Demographic and competition characteristics of the study sample.** | |
| --- | --- |
| **Variable** | **Description** |
| Sex | Male |
| Competition level | Senior international elite |
| Age eligibility | Male senior artistic gymnasts who met the FIG senior eligibility requirement (18 years or older during the year of competition) |
| Competition type | Olympic Games and World Championships |
| Number of competitions | 12 competitions: 3 Olympic Games and 9 World Championships |
| Competition period | 2013–2024 |
| Total finalist appearances | 580 |
| Apparatus events | Floor exercise, pommel horse, still rings, vault, parallel bars, and horizontal bar |
| **Note.** The competitions included three Olympic Games—2016 (Rio de Janeiro), 2020 (Tokyo), and 2024 (Paris)—and nine World Championships: 2013 (Antwerp), 2014 (Nanning), 2015 (Glasgow), 2017 (Montreal), 2018 (Doha), 2019 (Stuttgart), 2021 (Kitakyushu), 2022 (Liverpool), and 2023 (Antwerp). | |
