# Supplementary material for: Start order and medal outcomes: An analysis of apparatus finals in men’s artistic gymnastics
Source: PLoS One. 2026 Jun 18;21(6):e0351760. doi: 10.1371/journal.pone.0351760 (PMC13278440; doi:10.1371/journal.pone.0351760)
Supplement: S2 Table — This table provides the FIG Event IDs and direct URLs for the three Olympic Games and nine World Championships included in the present analysis. Each URL links to an official FIG/World Gymnastics event page. The official result files can be accessed from each event page by clicking “Show Files” under “Event Files.”. (DOCX) [file pone.0351760.s003.docx]

| **S2 Table. FIG / World Gymnastics event identifiers and direct URLs for competitions included in this study.** | | | | | |
| --- | --- | --- | --- | --- | --- |
| **No.** | **Competition** | **Year** | **Location** | **FIG Event ID** | **Direct URL** |
| 1 | WCH | 2013 | Antwerp | 5351 | <https://www.gymnastics.sport/site/events/detail.php?id=5351> |
| 2 | WCH | 2014 | Nanning | 5529 | <https://www.gymnastics.sport/site/events/detail.php?id=5529> |
| 3 | WCH | 2015 | Glasgow | 5346 | <https://www.gymnastics.sport/site/events/detail.php?id=5346> |
| 4 | OG | 2016 | Rio de Janeiro | 6405 | <https://www.gymnastics.sport/site/events/detail.php?id=6405> |
| 5 | WCH | 2017 | Montreal | 13465 | <https://www.gymnastics.sport/site/events/detail.php?id=13465> |
| 6 | WCH | 2018 | Doha | 13335 | <https://www.gymnastics.sport/site/events/detail.php?id=13335> |
| 7 | WCH | 2019 | Stuttgart | 14274 | <https://www.gymnastics.sport/site/events/detail.php?id=14274> |
| 8 | OG | 2020 | Tokyo | 15571 | <https://www.gymnastics.sport/site/events/detail.php?id=15571> |
| 9 | WCH | 2021 | Kitakyushu | 16634 | <https://www.gymnastics.sport/site/events/detail.php?id=16634> |
| 10 | WCH | 2022 | Liverpool | 15871 | <https://www.gymnastics.sport/site/events/detail.php?id=15871> |
| 11 | WCH | 2023 | Antwerp | 16194 | <https://www.gymnastics.sport/site/events/detail.php?id=16194> |
| 12 | OG | 2024 | Paris | 17041 | <https://www.gymnastics.sport/site/events/detail.php?id=17041> |
| **Note.** WCH = World Championships; OG = Olympic Games. This table provides the FIG Event IDs and direct URLs for the three Olympic Games and nine World Championships included in the present analysis. Each URL links to an official FIG / World Gymnastics event page. The official result files can be accessed from each event page by clicking “Show Files” under “Event Files.” | | | | | |
